# Dual G9A/EZH2 Inhibition Stimulates Antitumor Immune Response in Ovarian High-Grade Serous Carcinoma

**DOI:** 10.1158/1535-7163.MCT-21-0743

**Published:** 2022-02-07

**Authors:** Pavlina Spiliopoulou, Sarah Spear, Hasan Mirza, Ian Garner, Lynn McGarry, Fabio Grundland-Freile, Zhao Cheng, Darren P. Ennis, Nayana Iyer, Sophie McNamara, Marina Natoli, Susan Mason, Karen Blyth, Peter D. Adams, Patricia Roxburgh, Matthew J. Fuchter, Bob Brown, Iain A. McNeish

**Affiliations:** 1 Department of Surgery and Cancer, Ovarian Cancer Action Research Centre, Imperial College London, London, United Kingdom.; 2 Institute of Cancer Sciences, University of Glasgow, Glasgow, United Kingdom.; 3 Cancer Research UK Beatson Institute, Glasgow, United Kingdom.; 4 Sanford Burnham Prebys Medical Discovery Institute, San Diego, California.; 5 Department of Chemistry, Molecular Sciences Research Hub, Imperial College London, London, United Kingdom.

## Abstract

Ovarian high-grade serous carcinoma (HGSC) prognosis correlates directly with presence of intratumoral lymphocytes. However, cancer immunotherapy has yet to achieve meaningful survival benefit in patients with HGSC. Epigenetic silencing of immunostimulatory genes is implicated in immune evasion in HGSC and re-expression of these genes could promote tumor immune clearance. We discovered that simultaneous inhibition of the histone methyltransferases G9A and EZH2 activates the CXCL10–CXCR3 axis and increases homing of intratumoral effector lymphocytes and natural killer cells while suppressing tumor-promoting FoxP3^+^ CD4 T cells. The dual G9A/EZH2 inhibitor HKMTI-1–005 induced chromatin changes that resulted in the transcriptional activation of immunostimulatory gene networks, including the re-expression of elements of the ERV-K endogenous retroviral family. Importantly, treatment with HKMTI-1–005 improved the survival of mice bearing *Trp53^−/−^* null ID8 ovarian tumors and resulted in tumor burden reduction. These results indicate that inhibiting G9A and EZH2 in ovarian cancer alters the immune microenvironment and reduces tumor growth and therefore positions dual inhibition of G9A/EZH2 as a strategy for clinical development.

## Introduction

Despite ample evidence that the prognosis of patients with advanced ovarian high-grade serous carcinoma (HGSC) is strongly influenced by the immune microenvironment (1), current immunotherapies have failed to produce a meaningful survival benefit for patients (2). HGSC cells can evade immune responses by altering their epigenome, and targeting ovarian cancer epigenetics can reactivate cancer testis antigens (3), induce viral mimicry (4), and alter the tumor immune microenvironment and immune cell function (5). DNA methylation and histone deacetylation are two mechanisms that play a role in cancer immune evasion (6), and, although inhibitors of both DNA methylation and histone deacetylation are currently used in some hematologic malignancies, their use in solid malignancies has been limited due to toxicity and limited efficacy (7). More recently, histone methylation mediated by both G9A and EZH2 has been identified as an important pathway that influences the immune system in ovarian cancer and multiple other malignancies, including multiple myeloma and hepatocellular carcinoma (8–12).

Increased levels of the chemokines CXCL9, CXCL10, CXCL11, and CCL5 are all associated with an immune-reactive ovarian cancer microenvironment and improved patient prognosis (13). CXCL9, CXCL10, and CXCL11 are IFN-inducible and bind to CXCR3. The Cancer Genomic Atlas (TCGA) Research Network identified a subgroup of patients with HGSC with an activated CXCR3/CXCL9–11 pathway (14). Critically, when these chemokines are present at high concentrations within tumors, patients achieve a longer disease-free interval and overall survival (15). The primary role of these IFNγ-inducible chemokines is trafficking of activated CD8^+^, CD4^+^ T cells, and natural killer (NK) cells. In preclinical models of ovarian cancer, increased expression of CXCL10 can reduce tumor burden and ascites accumulation (16). CCL5 is also associated with T-cell infiltration and tumor control in other carcinomas (17). Coukos and colleagues recently showed that CCL5^hi^CXCL9^hi^ ovarian tumors are immunoreactive and responsive to immune checkpoint blockade, with tumor-derived CCL5 driving expression of CXCL9 from intratumoral immune cells, such as antigen-presenting cells, which in turn supports T-cell engraftment in the tumor (18). We reasoned that pharmacologic approaches to activate the CXCR3/CXCL9–11 pathway might be of therapeutic benefit in ovarian cancer.

Using a medium-throughput screening library of epigenetic compounds, we sought to discover epigenetic mechanisms that can augment immune responses in HGSC. We discovered that dual inhibition of G9A and EZH2 histone lysine methyltransferases induces potent release of lymphocyte chemotactic chemokines, including CXCL9, CXCL10, CXCL11, and CCL5, confirming these results in a panel of human cell lines and primary patient samples. We also showed that the dual G9A/EZH2 inhibitor HKMTI-1–005 (19) powerfully modified accessible chromatin in a syngeneic HGSC model, accompanied by transcriptional upregulation of immune pathways and, critically, substantial modulation of the tumor immune microenvironment. Importantly, we describe how G9A/EZH2 inhibition generated a significant influx of effector CD8^+^ T cells, NK cells, activated conventional type 1 dendritic cells (cDC1) while depleting tumors of CD4^+^ T regulatory cells. We observed a significantly extended survival of mice treated with HKMTI-1–005, indicating that G9A/EZH2 inhibition may provide a useful tool to overcome the poor immune reaction to ovarian cancer in patients.

## Materials and Methods

### Ethics statements

All *in vivo* experiments performed in mice were approved by the Animal Welfare & Ethics Review Body (AWERB) at the University of Glasgow and Imperial College London. Experiments were performed under the project license numbers 70/8645 at the Cancer Research UK Beatson Institute and project licenses 70/7997 and PA780D61A at Imperial College London. All experiments conformed to UK Home Office regulations under the Animals (Scientific Procedures) Act 1986, including Amendment Regulations 2012. Ascites from patients with ovarian cancer was collected and utilized under the auspices and ethical approval of the Imperial College Healthcare Tissue Bank (HTA license 12275, Research Ethics Committee number 17/WA/0161, Project ID R18060).

### *In vivo* syngeneic mouse model of ovarian cancer and cell lines

We utilized the ID8 syngeneic murine model (20) with bi-allelic *Trp53* deletions that we described previously (21). A total of 5 × 10^6^*Trp53^−/−^* ID8 cells/mouse were injected intraperitoneally in 6-week-old C57BL/6J female mice. At defined endpoint, ascites, intra-abdominal tumors (formed in omentum and porta hepatis) and spleens were collected (Supplementary Fig. S1). When no ascites was present, peritoneal cells were collected by lavage with 5 mL PBS. For survival experiments, humane endpoints included weight loss of 20% or more, ascites equivalent to full term pregnancy, reduced/slow activity, pale feet, and visible symptoms of distress such as hunching, piloerection, closed eyes, and isolation from cage mates.

HKMTI-1–005 (patent WO/2013/140148; ref. 19) was synthesized at Imperial College London and converted to HCl salt, which was used in the biological experiments. It was dissolved in DMSO for long term storage and reconstituted in 1% Tween/0.9% NaCl vehicle, just prior to injection, and given as twice daily intraperitoneal injections of 20 mg/kg. Mice were randomly assigned to a 2-week treatment with HKMTI-1–005 or vehicle alone (control) starting on day 21 following intraperitoneal cell inoculation. The investigators deciding on endpoint were blinded to the treatment administered.

Kuramochi and OVCAR-3 cells were obtained from Professor Sadaf Ghaem-Maghami (Imperial College London). OVCAR-4 were obtained from Dr Richard Camalier (NCI Frederick). All human cell lines were authenticated by 16 locus short tandem repeat profiling (Eurofins Genomics, January 2019) and tested weekly for mycoplasma infection (MycoAlert Mycoplasma Detection Kit (Lonza, LT01–318).

### Drug library screening

The drug library of epigenetic compounds was provided by Structural Genomic Consortium (Supplementary Table S1). 2×10^4^*Trp53^−/−^* ID8 cells/per well were seeded in 364-well black polypropylene, flat-bottom plates on day −1. On day 0, the drug library was added, along with 1 ng/mL of mouse IFNγ (Thermo Fisher Scientific, #PMC4031). After 72 hours, supernatant was transferred onto CXCL10 ELISA plates (R&D Systems DY466) using the JANUS G3 MDT automated workstation (Perkin Elmer) for downstream analysis (R&D Systems, DY466). Cell viability was measured by 4′,6-diamidino-2-phenylindole (DAPI) staining.

### Gene expression assays

RNA was extracted from cells with the Qiagen RNeasy protocol (Catalog No. 74004). Quality control and quantification were performed using the NanoDrop 2000 spectrophotometer (Thermo Fisher Scientific). RNA was aliquoted and stored at −80°C. cDNA synthesis was performed using high-capacity cDNA Reverse Transcription Kit from Thermo Fisher Scientific (4368814) and iTaq/universal probes mastermix (Bio-Rad, 1725131) was used for single-gene RT-qPCR reactions (Supplementary Table S2). Chemokine expression was quantified using RT^2^ Profiler PCR array for mouse chemokines/cytokines (Qiagen PAMM-150ZA, 330231) with data analysis performed using the Qiagen online tool PCR Array data analysis Web portal (https://www.qiagen.com/gb/resources/resourcedetail?id=20762fd2-8d75-4dbe-9f90-0b1bf8a7746b&lang=en). The chemokines tested, quality control, and normalization analysis are found in Supplementary Tables S3 to S5.

### Human ascites

After sterile collection, spheroids were captured on a 40 μm membrane and placed into T75 ultra-low attachment flasks (ULA, Corning, 3814) and cultured in advanced DMEM/F12 medium (Life Technologies, 12634010), supplemented with 10% autologous ascites, 10 mmol/L HEPES, 1× N-2 supplement (Thermo Fisher Scientific, 17502048), 1× serum-free B-27 supplement (Thermo Fisher Scientific, 17504044), 100 U/mL penicillin plus 100 μg/mL streptomycin (penicillin/streptomycin, Thermo Fisher Scientific, 15140–122), and 2 mmol/L L-glutamine (Thermo Fisher Scientific, 25030–081). Spheroids were allowed to grow for up to 72 to 96 hours after which they were dissociated and treated as a monolayer. Cells were stained for PAX8 and sequenced for *TP53* (Illumina Ampliseq) as detailed in Supplementary Materials and Methods. Patient details and cell line data are given in Supplementary Table S6.

### Flow cytometry

Fresh tumor deposits from the omentum and the porta hepatis of mice bearing *Trp53^−/−^* ID8 tumors were harvested. Tumor digestion was performed as described previously (22) modified with the use of collagenase (Sigma, C7657) and dispase (Gibco, 17105041). Tumor cells (20 × 10^6^/mL) were plated in 96-well V-bottomed plates followed by FcR II/III block [BD Biosciences, 553142, diluted 1:200 in FACS (0.5% FBS, 2 mmol/L EDTA in PBS) buffer]. Antibody details are given in Supplementary Table S7. Cells were fixed with 2% neutral-buffered formalin diluted in FACS buffer following addition of Zombie Yellow fixable viability dye (BioLegend, #423103, 1:200 in PBS).

For intracellular assessment of T-cell activity, 20 × 10^6^/mL tumor cells were plated in clear untreated U-bottom plates (SLS, 3879) after tumor digestion. After stimulation (PMA and ionomycin, eBioscience, 00–4970, 2 μL/mL, 1 hour), protein transport inhibitor cocktail (eBioscience, 00–4980, 2 μL/mL) was added. After 4 hours, cells were transferred to a V-bottom plate and stained for the membrane markers, viability dye Zombie Yellow, and fixed followed by intracellular staining using the intracellular staining permeabilization buffer (BioLegend, 421002). Samples were analyzed on a 3-LASER Cytek Aurora (Cytek Biosciences) cytometer and the software FlowJo 10.7.1. Only samples that reached a threshold of 200 events per sample were included in the quantitative analysis. The geometric mean fluorescence intensity (MFI) was calculated by subtracting by an average (minus FMO) fluorescence value from pooled samples from each individual test sample.

### RNA and ATAC sequencing

Frozen mouse tumors (≤10 mg) were homogenized in a Precellys homogenizer using ceramic beads at 2,000 × *g* for two pulses of 30 seconds. RNA was extracted from the lysate with the Qiagen RNeasy protocol (Catalog No. 74004). RNA with an RNA Integrity number (RIN) of >7 as measured in an Agilent 2200 TapeStation was used for downstream-sequencing analysis. Following ribosomal RNA depletion (NEBNext, E6350) from 250 ng total RNA, sequencing libraries were constructed using the NEBNext Ultra II Directional Library Prep Kit for Illumina (E7760S). Following QC (Agilent D5000 Screen Tape System) and quantification [Qubit dsDNA High-Sensitivity Assay Kit (Thermo Fisher Scientific, Q32854)], samples were sequenced [Nova6000 SP flow cell (Illumina) 50 bp PE, target 50 million read pairs per sample].

For the assay of transposase-accessible chromatin using sequencing (ATAC-seq), the published protocol Omni-ATAC (23) was optimized for *Trp53^−/−^* ID8 tumors. 20 mg tumor deposits were homogenized with a glass Dounce homogenizer. The lysate was then mixed in an iodixanol concentration gradient (25%–29%–35% concentration gradient, iodixanol Sigma-Aldrich, D1556) and centrifuged in a swinging bucket at 4,000 × *g* for 20 minutes. After centrifugation, 20,000 nuclei per sample were harvested from the nuclear band and treated with 100 nmol/L hyperactive transposase enzyme (Nextera Tagment DNA enzyme I #15027916) for 30 minutes at 37°C, shaking at 400 rpm, in an Eppendorf Thermomixer comfort incubator. The purified, transposed DNA was amplified using customized primers as published previously (24). After quality control (Agilent D5000 Screen Tape System), the amplified library was sequenced [Nova6000 S1 flow cell (Illumina), 50 bp PE].

Raw-sequencing reads were aligned to mouse genome version GRCm38.p4 (mm10) using the STAR aligner with default parameters (25). Raw counts were generated using the Rsubread package (26) and differentially expressed genes (DEG) were identified using the DESeq package (27). All analyses, statistical tests, and plots were generated in R version 3.3.3 unless specified otherwise. MultiQC was used to collate data across different programs (28). For functional annotation of DEGs, we used the Database for Annotation, Visualization and Integrated Discovery (DAVID) online Functional Annotation Tool (29) with access to Gene Ontology (GO; ref. 30), and KEGG (31) databases. For analysis of endogenous retroviruses (ERV), a mm10 annotation for mouse endogenous viral elements was obtained from the gEVE database (http://geve.med.u-tokai.ac.jp). During alignment, only primary alignments were taken into account, a method adapted by Haase and colleagues (32). For ATAC-seq methodology, the MACS2 tool was used to call peaks on all individual control and treatment samples (33). Immune cell composition was inferred using seq-ImmuCC (34)

### Data availability

All RNA-seq and ATAC-seq data are available in Supplementary Tables S8 to S11 and via ENA (https://www.ebi.ac.uk/ena/submit/sra/#home). Primary accession no. PRJEB44851, secondary accession no. ERP12894.

### Statistical analysis

Statistical analyses were performed in GraphPad Prism (v9.0.0). For mean comparisons between two groups, *t* test was used for populations with normal distribution and Mann–Whitney test for nonparametric distribution. One-way ANOVA was used for comparison of more than two groups. Matched-pair *t* test was used to compare mean values for patient samples. Log-rank test was used to compare differences in survival. When indicated, ROUT (*Q* = 1%) method was used to identify outliers.

## Results

### Combined G9A/EZH2 inhibition upregulates chemotactic chemokines *in vitro*

**Figure 1.**
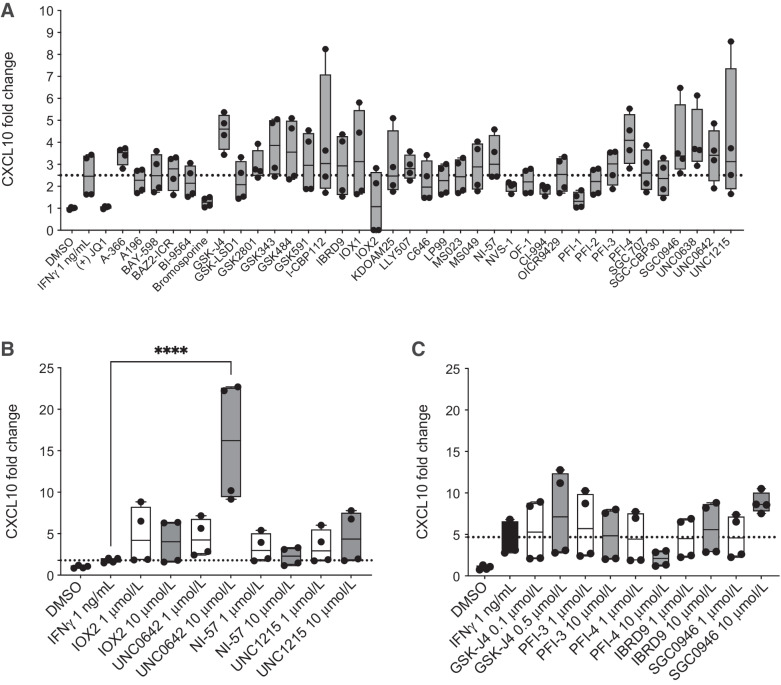
G9A inhibition upregulates CXCL10 in an ovarian cancer model. **A,** 2 × 10^3^*Trp53^−/−^* ID8 cells in 384-well plates were treated with the SGC drug library (all drugs were at a final concentration of 1 μmol/L apart from GSK-J4: 0.2 μmol/L) with 1 ng/mL of murine IFNγ. CXCL10 ELISA was performed on day 4. Box and whiskers show all values obtained from four technical replicates. Mean values were compared with IFNγ stimulation alone, using one-way ANOVA with Dunnett multiple comparisons test. The results were not statistically significant. **B** and **C**, CXCL10 protein fold change following treatment with 10 selected SGC library drugs (1 and 10 μmol/L apart from GSK-J4: 0.1 and 0.5 μmol/L). **B** and **C,** show data from separate experiments. Box and whiskers show all values obtained from four technical replicates. One-way ANOVA with Dunnett multiple comparison test was used to compare all mean values to IFNγ alone; statistically nonsignificant results are not shown (****, *P* < 0.0001).

We initially screened 38 epigenetic drugs for CXCL10 production by IFNγ stimulated *Trp53^−/−^* ID8 (21) cells using ELISA. We wished to identify chromatin modifying drugs that could enhance IFNγ-induced *Cxcl10* transcription. Although no statistically significant increases were observed in an initial single concentration screen (Fig. 1A), a two-dose re-screen was performed with 10 drugs that had caused a numerical increase in mean CXCL10 production and that covered a wide range of epigenetic targets (Fig. 1B). UNC0642, an inhibitor of G9A (EHMT2) and G9A-like protein (GLP; ref. 35), significantly upregulated CXCL10 production compared with IFNγ stimulation alone (mean fold change 16.1 ± 7.4 vs. 1.8 ± 0.3, *P* < 0.0001; Fig. 1B and C), at doses that were not cytotoxic (dose–response curves, Supplementary Fig. S2).

**Figure 2.**
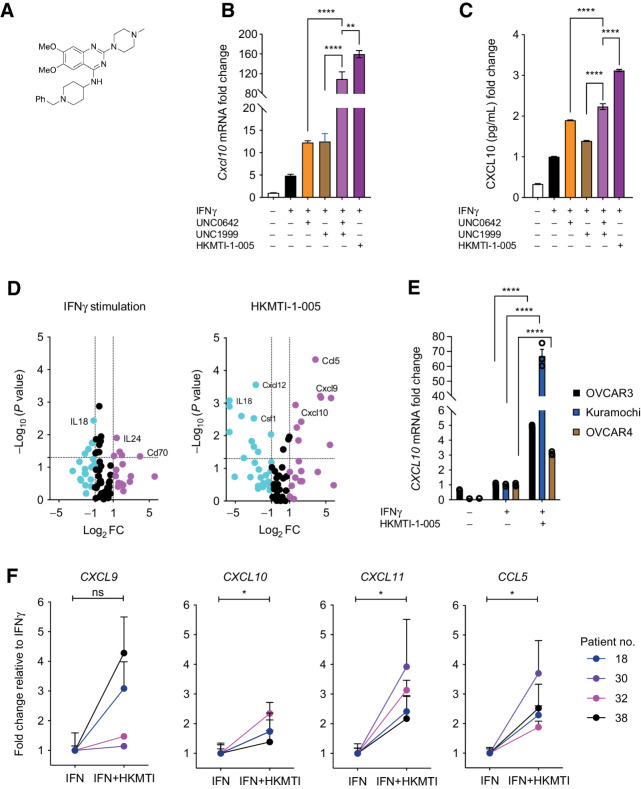
Dual inhibition of G9A/EZH2 upregulates chemotactic chemokines *in vitro* in mouse and human. **A,** Chemical structure of HKMTI-1–005. **B,***Cxcl10* mRNA fold change, normalized to *Gapdh* housekeeping gene, following treatment of *Trp53^−/−^* ID8 cells with IFNγ 1 ng/mL with or without UNC0642 5 μmol/L (G9A inhibitor), UNC1999 2 μmol/L (Ezh2 inhibitor), or HKMTI-1–005 6 μmol/L (dual G9A/EZH2 inhibitor) for 72 hours. Mean values across treatments were compared with ordinary one-way ANOVA with Tukey multiple comparison test. Bars represents mean ± SEM, *n* = 3 biological replicates. Doses for UNC0642 and UNC1999 differed from the initial screening (Fig. 1) following further optimization. **C,** CXCL10 protein fold change determined by ELISA following treatment as per **A**. Mean values across treatments were compared using one-way ANOVA with Dunnett multiple comparisons test. Bars represents mean ± SEM, *n* = 3 biological replicates. **D,** Expression of 84 chemokines and cytokines following treatment of *Trp53^−/−^* ID8 cells with IFNγ 1 ng/mL (left) with or without treatment with HKMTI-1–005 6 μmol/L (right) for 72 hours. The experiment was performed in technical triplicates. Magenta color: Log_2_FC ≥ 1, black color: −1 ≤ Log_2_FC ≤ 1 and cyan color: Log_2_FC ≤ −1. Gene list is found on Supplementary Table S3, quality control and automatic normalization analysis in Supplementary Tables S4 and S5, respectively. **E,***CXCL10* mRNA change following treatment of OVCAR3, Kuramochi and OVCAR4 cell lines with IFNγ 1 ng/mL or HKMTI-1–005 10 μmol/L plus IFNγ 1 ng/mL for 72 hours. ΔΔ*C*_T_ values were normalized to control *ACTB* housekeeping gene *C*_T_ values. Mean values across treatments were compared by ordinary one-way ANOVA with Tukey multiple comparison test and only comparisons between HKMTI-1–005 10 μmol/L plus IFNγ versus IFNγ alone are shown. Each bar represents mean ± SEM, *n* = 3 biological replicates. **F,***CXCL9*, *CXCL10*, *CXCL11*, and *CCL5* expression in four human ascites derived cultures. Three with ovarian HGSC and one with grade 2 to 3 endometrioid ovarian carcinoma. Each dot represents the mean ± SEM of three technical replicates for each patient. Δ*C*_t_ values were normalized to *ACTB* housekeeping gene. Data represent fold change relative to IFNγ. Mean values were compared using paired *t* tests (****, *P* < 0.0001; ***, *P* < 0.001; **, *P* < 0.01; *, *P* < 0.05; ns, nonsignificant).

As G9A can cooperate closely with Enhancer of Zeste homolog 2 (EZH2; ref. 36), we combined UNC0642 with the EZH2 inhibitor UNC1999, the latter chosen based on prior published data (37) and drug availability. We also evaluated HKMTI-1–005 (Fig. 2A), the first described dual G9A/EZH2 inhibitor (19), which, in contrast to other EZH2 inhibitors, has a peptide substrate competitive mechanism. The combination of UNC0642 and UNC1999 induced a greater increase of both *Cxcl10* mRNA and CXCL10 protein than either drug alone (mRNA mean fold change 109.4 ± 25.0 vs. 12.3 ± 0.67 vs. 12.49 ± 3.1, *P* < 0.0001; protein mean fold change 2.23 ± 0.07 vs. 1.9 ± 0.01 vs. 1.4 ± 0.01, *P* < 0.0001, Fig. 2B and C). At doses that reduced repressive histone marks (Supplementary Fig. S3) and that were not significantly cytotoxic (Supplementary Fig. S4), HKMTI-1–005 induced stronger upregulation of *Cxcl10* than either methyltransferase inhibitor alone (mean fold change 159.6 ± 12.5 vs. 12.3 ± 0.67 vs. 12.49 ± 3.1, *P* < 0.0001, Fig. 2B), as well as combination treatment with the two single inhibitors (mean fold change 159.6 ± 12.5 vs. 109.4 ± 25.0, *P* = 0.001, Fig. 2B). HKMTI-1–005 treatment also resulted in higher CXCL10 protein production than the individual inhibitors given alone (mean fold change 3.1 ± 0.03 vs. 1.9 ± 0.01 vs. 1.4 ± 0.01, *P* < 0.0001) and in combination (mean fold change 3.1 ± 0.03 vs. 2.2 ± 0.1, *P* < 0.0001, Fig. 2C).

An 84 chemokine/cytokine gene expression array confirmed that dual G9A/EZH2 inhibition with HKMTI-1–005 had a potent effect on chemokine expression *in vitro*. Specifically, it upregulated *Cxcl10* 3-fold (*P* = 0.001), *Cxcl9* 22-fold (*P* = 0.0006), and *Ccl5* 14-fold (*P* < 0.0001), compared with IFNγ stimulation alone (Fig. 2D). In contrast, chemokines secreted upon cell death, such as *IL1* or *IL18*, were not increased (*IL1a* fold-change 0.43, *P* = 0.34; *IL1b* fold-change 0.61, *P* = 0.30; and *IL18* fold-change 0.02, *P* < 0.0001). This implies that the upregulation of the CXCR3-binding chemokines CXCL9, CXCL10, and CCL5 is not a cell death-associated stress response. HKMTI-1–005 treatment also upregulated *CXCL10* transcription in established human HGSC cell lines, including OVCAR3 (fold change 4.9, *P* < 0.0001), OVCAR4 (fold change 3.1, *P* < 0.0001), and Kuramochi (fold change 66.9, *P* < 0.0001), when compared with IFNγ stimulation alone (Fig. 2E). These lines were selected for their resemblance to HGSC at phenotypic, genomic, and copy-number level (38). Importantly, treatment of ascites-derived primary cells from patients (three HGSC, one grade 2–3 endometrioid carcinoma) with HKMTI-1–005 significantly increased *CXCL10* (*P* = 0.028), *CXCL11* (*P* = 0.017), and *CCL5* (*P* = 0.027) mRNA levels, compared with IFNγ stimulation alone (Fig. 2F), demonstrating that dual G9A/EZH2 inhibition may have an immunostimulatory effect in the tumor microenvironment (TME) in patients with ovarian carcinoma.

### Dual G9A/EZH2 inhibition alters transcription and chromatin conformation *in vivo*

**Figure 3.**
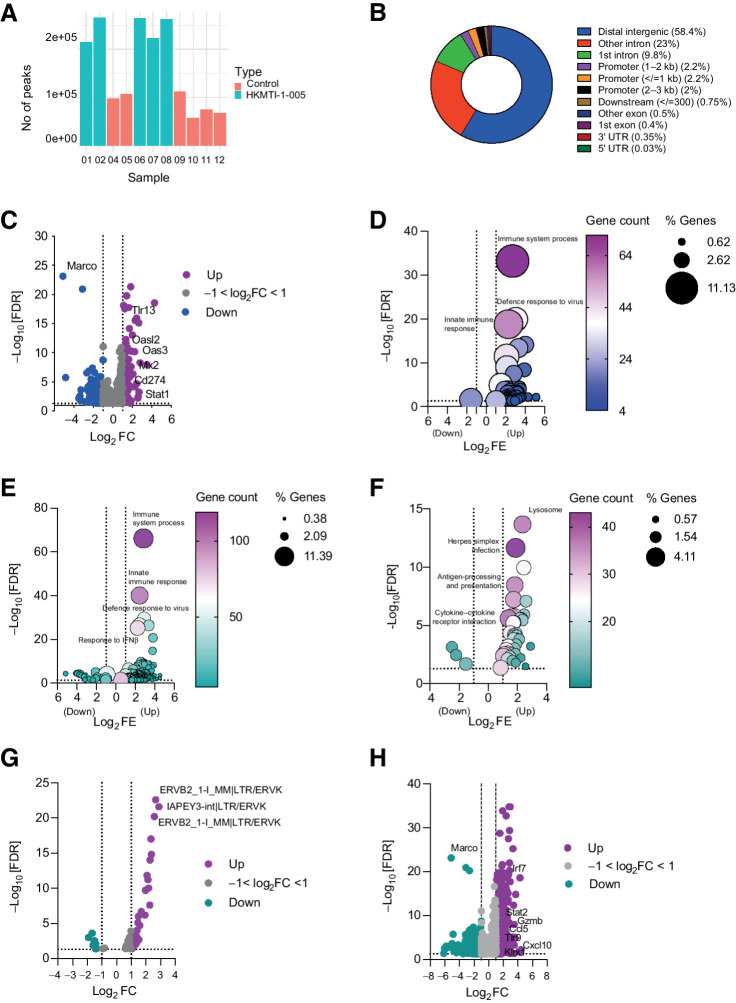
ATAC-seq and RNA-seq on murine omental tumor deposits. **A,** Number of ATAC-seq peaks for control (*n* = 6) and HKMTI-1–005 samples (*n* = 5), before applying filtration criteria. One HKMTI-1–005 sample did not yield enough sequencing reads and was removed from analysis. **B,** Distribution of ATAC-seq peaks across the genome. **C,** Overlap of genes with ATAC-seq peaks showing increased chromatin accessibility that were also differentially expressed (*n* = 1,106) by RNA-seq following HKMTI-1–005 treatment *in vivo* (HKMTI-1–005 *n* = 7, control *n* = 7). FC, fold change. Purple color: Log_2_FC ≥ 1, gray color: −1 < Log_2_FC ≤ 1, and blue color: Log_2_FC ≤ −1. **D,** Biological processes (BP) sub-ontology for 1,053 genes from **C** that overlapped with gene expression signatures from DAVID online Functional Annotation Tool. Gene count denotes the number of genes found to overlap with genes within the respective signature and the dot size represents the percentage of these genes within the signature. FE, fold enrichment. **E** and **F**, DEG following HKTMI-1–005 classified by BP and KEGG sub-ontologies, respectively. Gene count denotes the number of genes found to overlap with genes within the respective signature and the dot size represents the percentage of these genes within the signature. **G,** Volcano plot showing differentially expressed ERVs, following HKMTI treatment (*n* = 7) versus control (*n* = 7). Purple color: Log_2_FC ≥ 1, gray color: −1 < Log_2_FC ≤ 1 and green color: Log_2_FC ≤ −1. **H,** Volcano plot showing individual DEG following HKMTI treatment (*n* = 7) versus control (*n* = 7) by RNA sequencing with an FDR <0.05. Purple color: Log_2_FC ≥ 1, gray color: −1 < Log_2_FC ≤ 1 and green color: Log_2_FC ≤ −1. Cellular response to IFNβ (GO:0035458, FDR = 7.65e−15) and response to virus (GO:0009615, FDR = 4.32e−14).

We hypothesized that altered chromatin accessibility induced by G9A/EZH2 inhibition could explain the changes in gene expression. To investigate this, we used the ATAC-seq and RNA-seq on tumors harvested after 14 days of HKMTI-1–005 treatment. ATAC-seq showed more peaks representing areas of open chromatin in the HKMTI-1–005-treated samples compared with controls (Fig. 3A), with most peaks in intergenic regions (58.4%). Approximately 33% of peaks were intronic and 6.5% were in promoter regions (Fig. 3B). Peaks were present in genes involved in the activation pathways for *Cxcl9*, *Cxcl10*, and *Ccl5*, including *Stat1*, *Irf1*, NF-κB p105 subunit (*Nfkb1*), and inhibitor of NF-κB kinase subunit β (*Ikbkb*). We found a statistically significant overlap of 1,106 genes in common between DEG identified by RNA-seq and those in an euchromatin state identified by ATAC-seq (Fig. 3C). Among these were the Toll-like receptor *Tlr13* (Log_2_FC 2.9, FDR = 7.42e−19), the IFN pathway mediator *Stat1* (Log_2_FC 1.01, FDR = 4.1e−03), *Cd274*, which encodes PD-L1 (Log_2_FC 1.39, FDR = 8.99e−06), and genes involved in antiviral response, such as *Mx2* (Log_2_FC 1.86, 2.43e−08) and *Oas3* (Log_2_FC 2.2, FDR = 6.3e−11; Fig. 3C). Using the DAVID functional annotation tool, we found that the most statistically significant upregulated genes with open chromatin belonged to signatures categorized as immune system process (GO:0002376, FDR = 6.25e−34), defense response to virus (GO:0051607, FDR = 1.31e−20), and innate immune response (GO:0045087, FDR = 1.58e−19; Fig. 3D). Other significantly upregulated pathways were cellular response to IFNβ (GO:0035458, FDR = 7.65e−15) and response to virus (GO:0009615, FDR = 4.32e−14). More widely, treatment with HKMTI-1–005 significantly altered the transcriptome of tumors *in vivo.* The most significantly upregulated biological processes sub-ontology pathway in the HKMTI-1–005-treated tumors was the immune pathway immune system process (GO:0002376, FE 7.1, FDR = 7.43e−67), with more than 10% DEGs overlapping with genes in this pathway (Fig. 3E). Innate immune response (GO:0045087, FE 5.37, FDR = 9.55e−41) and defense response to virus (GO:0051607, FE 7.4, FDR = 3.12e−30) were also significantly upregulated (Fig. 3E). Fewer pathways were downregulated following HKTMI-1–005. Among those were positive regulation of glucose metabolic process (GO:0010907, FE 13.6, FDR = 1.82e−05), positive regulation of lipid metabolic process (GO:0045834, FE 16.6, FDR = 3.21e−05), and negative regulation of gluconeogenesis (GO:0045721, FE 11.9, FDR = 5.02e−05). Analysis using the KEGG database also revealed immune pathways being significantly enriched after treatment, such as antigen processing and presentation (mmu04612, FE 5.4, FDR = 1.11e−10), natural killer cell mediated cytotoxicity (mmu04650, FE 3.3, FDR = 6.45e−05), and cytokine–cytokine receptor interaction (mmu04660, FE 2.6, FDR = 2.51e−06; Fig. 3F).

Because we observed an upregulation of the defense response to virus pathway, we analyzed the differential expression of endogenous retroviruses (ERV) following HKMTI-1–005 treatment. These ancient transposable elements are epigenetically silenced under homeostatic conditions (39) but can potentiate antitumor immunity if transcriptionally active (4). 51 ERVs were differentially expressed at the 5% FDR threshold following HKMTI-1–005 treatment, of which 39 were upregulated (Fig. 3G), including the IAP ERVK elements IAPEY3-int|LTR/ERVK (log_2_FC 2.89; FDR = 2.40e−22) and ERVB2 ERVK (log_2_FC 2.67; FDR = 2.41e−23). In contrast, almost all of the downregulated retrotransposons belonged to the ERV1 class, including MuRRS4-int|LTR/ERV1 (log_2_FC −1.92, FDR < 0.001) and MURVY-int|LTR/ERV1 (log_2_FC −1.6, FDR < 0.001).

At the individual gene level, a total of 1,146 genes were upregulated and 733 genes downregulated following HKMTI-1–005 treatment (Fig. 3H). Among the upregulated genes were *Cxcl10* (Log_2_FC 1.69, FDR < 0.001), *Cxcl11* (Log_2_FC 1.19, FDR < 0.001), and *Ccl5* (Log_2_FC 1.84, FDR = 8.33e−08). A number of other immune-stimulatory genes were also upregulated, including the gene encoding granzyme B (*GzmB*; Log_2_FC 2.74, FDR = 3.49e−09) and *Klrk1* (Log_2_FC 1.15, FDR = 0.016), which encodes NKG2D, the major NK- and T-cell receptor for recognition and elimination of tumor cells. Moreover, *Stat2* (Log_2_FC 1.36, FDR = 1.61e−10) and *Tlr9* (Log_2_FC 1.21, FDR = 5.00e−05), integral parts of type I IFN-mediated responses, were also upregulated. Other type I IFN system mediators were upregulated with treatment, such as *Irf7* (Log_2_FC 2.28, FDR = 1.26e−19), *Irf9* (Log_2_FC 0.68, FDR = 1.79e−05), and *Irf5* (Log_2_FC 0.41, FDR = 0.02), as well as type I IFN inducible genes including *Oasl1* (Log_2_FC 1.87, 3.61e−19), *Oas2* (Log_2_FC 1.94, 5.79e−11), and *Oas3* (Log_2_FC 2.22, 6.30e−11), which are all involved in the antiviral defense gene network (40). *Olfr732* showed the largest reduction (Log_2_FC -6.13, FDR = 0.038), although its relation to cancer is unclear. The most statistically significant reduction occurred in Macrophage Receptor with Collagenous Structure *Marco* (Log_2_FC -5.12, FDR = 7.57e−24), which encodes a pattern-recognition receptor of the class A scavenger receptor family, expressed in tumor-associated macrophages (Fig. 3G). ATAC-seq confirmed that *Marco* had reduced chromatin accessibility (Fig. 3C), indicating HKMTI-1–005 may also act on tumor-associated macrophages.

### Dual G9A/EZH2 inhibition prolongs survival *in vivo*

**Figure 4.**
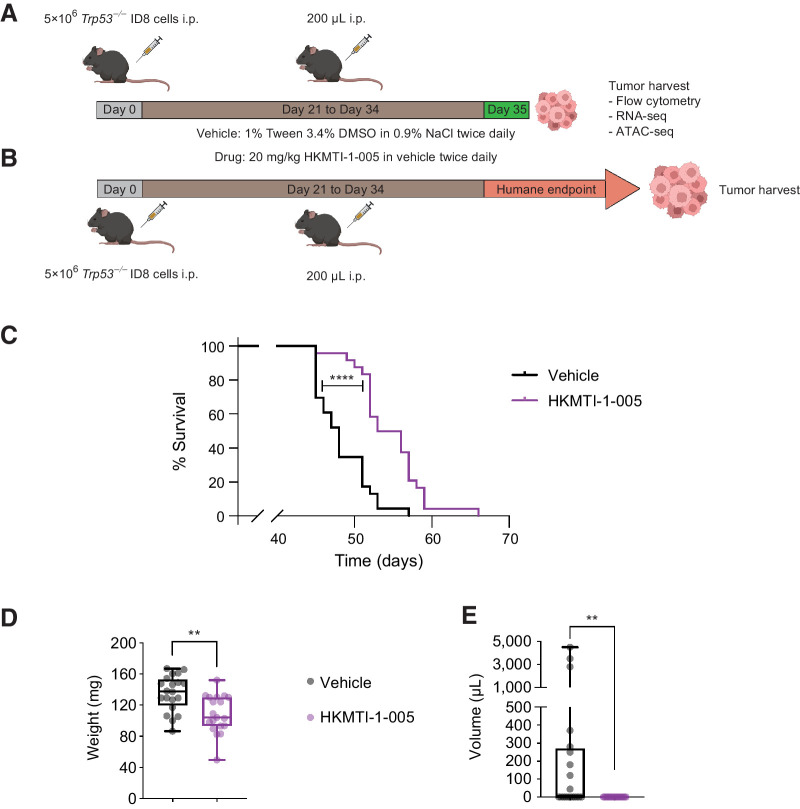
Dual G9A/EZH2 inhibition inhibits tumor growth and prolongs survival in a mouse ovarian cancer model. **A,** Experimental design for mechanism experiments. Mice bearing intraperitoneal *Trp53*^−/−^ID8 cells were treated with either HKMTI-1–005 (20 mg/kg i.p. twice a day) or vehicle (1% Tween/3.4% DMSO in 0.9% NaCl i.p. twice a day) for 14 days starting on day 21, followed by omental and porta hepatic deposits harvest/weighting, measuring ascites and immunophenotyping by flow cytometry immediately after the end of treatment. Image created with BioRender.com. **B,** Experimental design for efficacy experiments. Mice were treated as per **A**, but treatment was followed by observation until mice reached humane survival endpoint. **C,** Kaplan–Meier survival curves for mice treated with vehicle (*n* = 24) or 20 mg/kg HKMTI-1–005 (*n* = 24) as per schedule on B. Median survival was 48 days for vehicle versus 54.5 days for HKMTI-1–005, *P* < 0.0001). Curves were compared using the Log-rank (Mantel–Cox) test, ****, *P* < 0.0001. Experiment was performed twice with *n* = 12 per cohort for each experiment. **D,** Whole tumor weight (including both porta hepatis and omental tumor deposits) and **E** ascites volume for mice treated with either vehicle (*n* = 20) or HKMTI-1–005 (*n* = 20) as per schedule in **A**; comparisons were made using unpaired *t* test for whole tumor burden and Mann–Whitney test for ascites volume (**, *P* < 0.01). Experiment was performed twice with *n* = 10 per cohort for each experiment.

We next wanted to understand if modulating chromatin accessibility and stimulating gene expression, most importantly of chemokines associated with T- and NK-cell infiltration, could alter tumor growth and the immune response *in vivo* (Fig. 4A and B). HKMTI-1–005 treatment resulted in a prolongation of median survival (48 days vs. 54.5 days, *P* < 0.0001; HR, 0.33; 95% CI, 0.17–0.64; Fig. 4C), a reduction of tumor weight at the end of treatment (135 mg ± 5.2 vs. 108 mg ± 5.6, *P* = 0.001, Fig. 4D) and completely abrogated the development of ascites in this model (602 μL ± 297 μL vs. 0 μL, *P* = 0.0012, Fig. 4E). There were no toxicity signals observed throughout treatment and no significant weight difference between treatment groups (Supplementary Fig. S5).

### Dual G9A/EZH2 inhibition alters immune composition *in vivo* in tumor and peritoneal cavity

**Figure 5.**
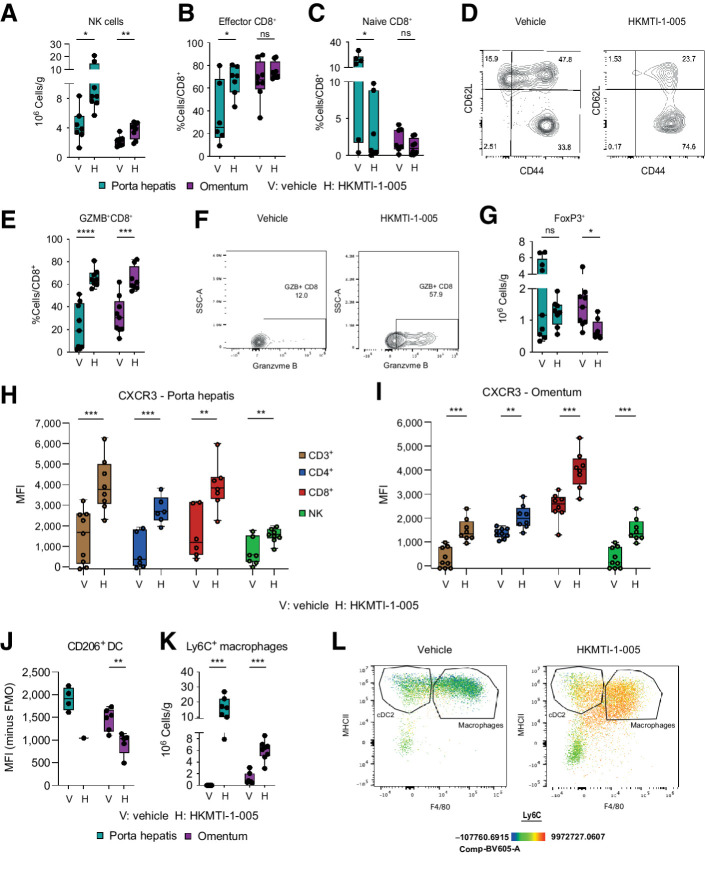
Dual G9A/EZH2 inhibition changes intratumoral immune cell composition in a mouse ovarian cancer model. **A,** Quantitative result for NK cells (CD3^−^ DX5^+^) cells in porta hepatis deposits with vehicle (*n* = 7) versus HKMTI-1–005 (*n* = 8) treatment and in the omental deposits in mice treated with vehicle (*n* = 9) versus HKMTI-1–005 (*n* = 8) treatment from mice bearing intraperitoneal *Trp53*^−/−^ ID8 cells were treated with either HKMTI-1–005 or vehicle as per Fig. 4A. Significance was tested using an unpaired *t* test. **B,** Percentage of effector CD8^+^ cells (CD44^+^CD62L^−^) within the total CD8^+^ population in porta hepatis deposits and omental deposits in mice treated with vehicle (*n* = 6 and 8, respectively) versus HKMTI-1–005 (*n* = 7 and 8, respectively) treatment. Significance was tested using an unpaired *t* test. **C,** Percentage of naïve CD8^+^ (CD44^−^ CD62L^+^) within the total CD8^+^ population in porta hepatis deposits and omental deposits in mice treated with vehicle (*n* = 6 and 8, respectively) versus HKMTI-1–005 (*n* = 7 and 8, respectively) treatment. Significance was tested using an unpaired *t* test (porta hepatis) and Mann–Whitney test (omental deposit). **D,** Representative contour plot from one of the omental deposits for effector and naïve CD8^+^ cells, in mice treated with vehicle versus HKMTI-1–005 treatment. **E,** Percentage of granzyme-B (GZMB^+^) CD8^+^ cells, following stimulation, within the total CD8^+^ population in porta hepatis deposits and omental deposits from mice treated with vehicle (*n* = 9 and 8, respectively) versus HKMTI-1–005 (*n* = 9 and 8, respectively) treatment. Statistical significance was tested by unpaired *t* test. **F,** Representative contour plot showing (GZMB^+^) CD8^+^ cells from one of the omental deposits from **E**. **G,** Quantitative result for T regulatory CD4^+^ cells (FoxP3^+^ CD4^+^) cells in porta hepatis deposits and omental deposits with vehicle (*n* = 9 and 8, respectively) versus HKMTI-1–005 (*n* = 9 and 8, respectively) treatment. Unpaired *t* test (porta hepatis deposits) and Mann–Whitney test (omental deposits). **H,** CXCR3 MFI on CD3^+^ (*n* = 9 vehicle and *n* = 8 HKMTI-1–005), CD4^+^ (*n* = 6 vehicle and *n* = 6 HKMTI-1–005), CD8^+^ (*n* = 6 vehicle and *n* = 7 HKMTI-1–005), and NK cells (*n* = 7 vehicle and *n* = 8 HKMTI-1–005) in the porta hepatis deposits. Statistical significance was tested by unpaired *t*-test. **I,** CXCR3 MFI on CD3^+^ (*n* = 9 vehicle and *n* = 8 HKMTI-1–005), CD4^+^ (*n* = 9 vehicle and *n* = 8 HKMTI-1–005), CD8^+^ (*n* = 8 vehicle and *n* = 8 HKMTI-1–005), and NK cells (*n* = 9 vehicle and *n* = 8 HKMTI-1–005) in omental deposits. Unpaired *t* test. **J,** CD206 MFI on cDC1 dendritic cells (CD11b^−^MHCII^+^CD11c^+^) in porta hepatis deposits (*n* = 4 vehicle and *n* = 1 HKMTI-1–005, statistics not performed as *n* < 3) and omental deposits (*n* = 6 vehicle and *n* = 5 HKMTI-1–005). Statistical significance was tested by unpaired *t* test. **K,** Ly6C^+^ macrophages (CD11b^+^MHCII^+^F4/80^+^) in porta hepatis and omentum deposits with vehicle (both *n* = 7) versus HKMTI-1–005 (*n* = 7 and 8, respectively) treatment. Statistical significance was tested by the Mann–Whitney test. **L,** Representative flow cytometry plot with pseudocolour heatmap showing Ly6C^+^ macrophages from a representative omental deposit from **K.** cDC2^+^ cells were subsequently gated on a CD11c+ (****, *P* < 0.0001; ***, *P* < 0.001; **, *P* <0.01; *, *P* < 0.05; ns, nonsignificant). Error bars represent SEM.

We hypothesized that the transcriptomic changes induced by HKMTI-1–005 could alter immune cell infiltration *in vivo*. We thus examined the effect of 14 days of HKMTI-1–005 treatment using two separate sites of disease in all analyses (Fig. 4A). Although cell composition inferred from RNA-seq data did not change significantly (Supplementary Fig. S6), flow cytometry showed significantly increased numbers of tumor-infiltrating NK cells (porta hepatis 3.8 × 10^6^ ± 0.8 vs. 8.2 × 10^6^ ±1.8 cells/g, *P* = 0.01; omentum 2.2 × 10^6^ ± 0.23 vs. 3.6 × 10^6^ ± 0.39 cells/g, *P* = 0.0067, Fig. 5A). Moreover, the effector CD44^+^CD62L^−^ CD8^+^ cytotoxic T-cell population was significantly increased in the porta hepatis deposit (37.1% ± 11.5% vs. 66.7% ± 4.9% effector CD8^+^/total CD8^+^, *P* = 0.03) with a similar, but statistically nonsignificant, trend in the omental deposit (67.2% ± 6.1% vs. 75.0% ± 2.1%, *P* = 0.27). Similarly, the naïve CD44^−^CD62L^+^ CD8^+^ T-cell population was decreased in the porta hepatis (14.1% ± 4.5% vs. 3.2% ± 1.6% naive CD8^+^/total CD8^+^, *P* = 0.03; Fig. 5B–D). In addition, granzyme-B^+^ CD8^+^ cells were significantly more frequent following treatment (porta hepatis 22.1% ± 6.6% vs. 65.7% ± 2.7% GZM-B^+^CD8^+^/total CD8^+^, *P* < 0.0001; omentum 32.3 ± 5.4% vs. 64.59 ± 3.8% GZM-B^+^CD8^+^/total CD8^+^, *P* = 0.0002; Fig. 5E and F), further indicating activation of effector CD8^+^ cells. These changes were accompanied by a decrease in the immunosuppressive FoxP3^+^ regulatory CD4^+^ population, mainly in omentum (1.2 × 10^6^ ± 0.16 vs. 0.5×10^6^ ± 0.10 cells/g, *P* = 0.014), with a statistically nonsignificant decrease in the porta hepatis (2.9 × 10^6^ ± 0.92 vs. 1.2 × 10^6^ ± 0.15 cells/g, *P* = 0.114; Fig. 5G). Expression of CXCR3 was increased on all lymphoid subsets in both tumor deposits, complementing our *in vitro* data (porta hepatis 1,581 ± 504.3 vs. 3,899 ± 432.3 MFI on CD8^+^ cells, *P* = 0.0049; omentum 2,485 ± 204.0 vs. 4,012 ± 273.6 MFI on CD8^+^ cells, *P* = 0.0005, Fig. 5H and I).

HKMTI-1–005 treatment decreased expression of CD206 on cDC1 (omental tumor 1,456 ± 100.5 vs. 925.8 ± 113.6 MFI, *P* = 0.006), a marker mainly associated with induction of T-cell tolerance (Fig. 5J; ref. 41). CD206 was also downregulated on macrophages in the omental deposit (5,220 ± 508 vs. 7,620 ± 626 MFI, *P* = 0.01; Supplementary Fig. S7), further supporting the hypothesis that G9A/EZH2 inhibition can block steps that lead to immunosuppression (42). HKMTI-1–005 treatment also resulted in a profound increase of Ly6C^+^ macrophages (porta hepatis 0 × 10^6^ vs. 16.21 × 10^6^ ± 2.56 cells/g, *P* = 0.0006; omentum 0.64 ± 0.38 × 10^6^ vs. 6.32 × 10^6^ ± 0.59 cells/g, *P* = 0.0006, Fig. 5K and L). Ly6C is a marker mostly expressed by precursors of tumor associated macrophages (TAM) and is thought to be downregulated as these precursors differentiate into TAMs. IHC staining confirmed similar trends in the omentum with regards to NK and FoxP3^+^ cell populations (Supplementary Fig. S8).

**Figure 6.**
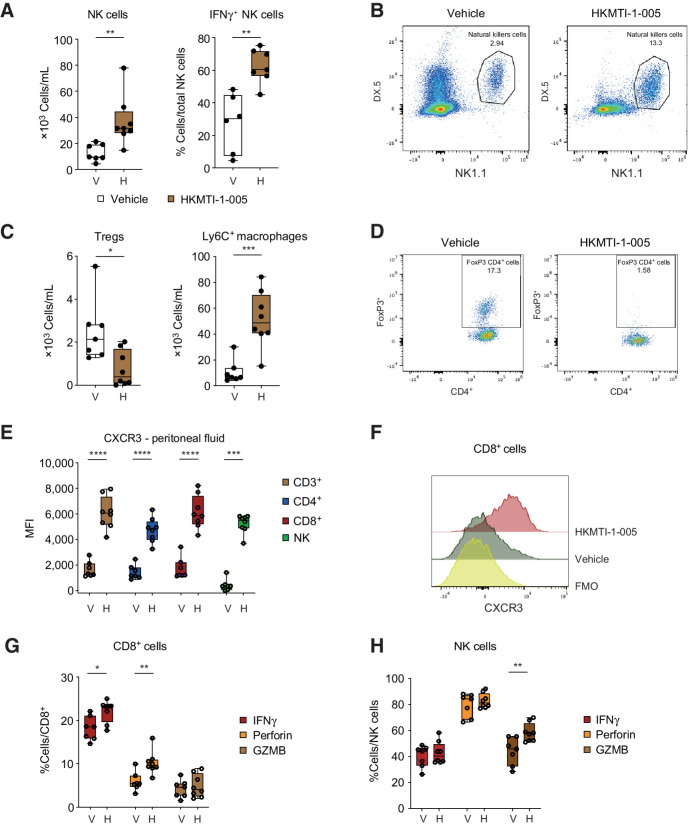
Dual G9A/EZH2 inhibition changes peritoneal cavity immune cell composition and chemokine milieu in the spleen in a mouse ovarian cancer model. **A,** (Left) Density of NK cells (CD3^−^ NK1.1^+^DX5^+^) in the peritoneal fluid with vehicle (*n* = 7) versus HKMTI-1–005 (*n* = 8) treatment from mice bearing intraperitoneal Trp53^−/−^ ID8 cells as per Fig. 4A. Statistical significance was tested by unpaired *t* test. (Right) Percentage IFNγ^+^ NK cells in peritoneal fluid from mice treated with vehicle (*n* = 6) versus HKMTI-1–005 (*n* = 7) treatment. Statistical significance was tested by unpaired *t* test. **B,** Representative flow cytometry plot from **A**. **C,** (Left) Density of regulatory T cells (CD4^+^FoxP3^+^) in peritoneal fluid from mice treated with vehicle (*n* = 7) versus HKMTI-1–005 (*n* = 8) treatment. Statistical significance was tested by unpaired *t* test. (Right) Density of Ly6C^+^ cells in the peritoneal fluid from mice treated with vehicle (*n* = 7) versus HKMTI-1–005 (*n* = 8) treatment. Statistical significance was tested by unpaired *t* test. **D,** Representative flow cytometry plot from **D**. **E,** CXCR3 MFI on CD3^+^ (*n* = 7 vehicle and *n* = 8 HKMTI-1–005), CD4^+^ (*n* = 7 vehicle and *n* = 8 HKMTI-1–005), CD8^+^ (*n* = 7 vehicle and *n* = 8 HKMTI-1–005), and NK cells (*n* = 7 vehicle and *n* = 8 HKMTI-1–005) in the peritoneal fluid. Unpaired *t* test was used to compare all mean values except NK-cell comparison where the Mann–Whitney test was used. **F,** Representative MFI histogram on one sample from **E**; FMO, fluorescence-minus-one. **G** and **H**. Percentage of IFNγ, perforin, and GZMB+ CD8^+^ cells (**G**) and NK cells (**H**) within their respective overall populations in spleen, following *ex vivo* stimulation with PMA and ionomycin and protein transport inhibitors brefeldin and monensin in mice treated with either vehicle (*n* = 7) versus HKMTI-1–005 (*n* = 8) treatment. Statistical significance was tested by unpaired *t* test (****, *P* < 0.0001; ***, *P* < 0.001; **, *P* < 0.01; *, *P* < 0.05; ns, nonsignificant). Error bars represent SEM.

The peritoneal cavity is an important site for the transcoelomic spread of HGSC with many patients developing ascites, which is rich in both tumor and immune cells. G9A/EZH2 blockade increased the absolute number of peritoneal NK cells (12.8 × 10^3^ ± 2.45 vs. 36.9 × 10^3^ ± 6.66 cells/mL, *P* = 0.006), a higher percentage of which were IFNγ^+^, indicative of an active antitumor response (27.5% ± 7.29 vs. 62.5% ± 3.97 IFNγ^+^NK cells/total NK cells, *P* = 0.001; Fig. 6A and B). As with the solid tumor deposits, depletion of T regulatory cells in the peritoneal cavity was observed (2.5 × 10^3^ ± 0.55 vs. 0.7 × 10^3^ ± 0.29 cells/mL, *P* = 0.01) and an increase in Ly6C^+^ macrophages (10.7 × 10^3^ ± 3.4 vs. 51.6 × 10^3^ ± 7.62 cells/mL, *P* = 0.0005; Fig. 6C and D). Furthermore, mirroring our intratumoral findings, the expression of CXCR3 was significantly increased on all lymphoid cell subpopulations (1,746 ± 318 vs. 6,144 ± 460 MFI on CD8^+^ cells, *P* < 0.0001; Fig. 6E and F), suggesting that this response is driven by the CXCL10 axis.

Finally, we analyzed the spleens of mice following G9A/EZH2 blockade to determine if treatment could potentiate an adaptive T-cell response in this important secondary lymphoid organ. Interestingly, we found that spleens treated with HKMTI-1–005 contained a higher percentage of CD8^+^ cells containing intracellular IFNγ (18.1% ± 1.04% vs. 22.1% ± 0.9% IFNγ^+^CD8^+^/total CD8^+^ cells, *P* = 0.012) and perforin (5.9% ± 0.8% vs. 10.2% ± 0.9% perforin positive CD8^+^/total CD8^+^ cells, *P* = 0.004, Fig. 6G). We also observed a higher percentage of NK cells containing granzyme-B (43.9% ± 4.0% vs. 58.4% ± 2.5% GZMB positive NK/total NK cells, *P* = 0.007, Fig. 6H). This provided further evidence that G9A/EZH2 blockade drives an antitumoral response, not only directly in the tumor deposits but also more systemically.

## Discussion

The drivers of the immune microenvironment in ovarian cancer remain unclear, although the extent of immune cell infiltration is strongly prognostic (13). Here we investigated whether modulation of epigenetic pathways could augment immune cell infiltration in HGSC, the commonest subtype of ovarian cancer in light of previous data suggesting that epigenetic mechanisms could underpin immune evasion in ovarian cancer (4, 43).

Using a mouse model that faithfully reproduces HGSC peritoneal dissemination, established cell lines, and ascites-derived primary cell cultures, we show that dual blockade of the histone methyltransferases G9A and EZH2 reprogrammed the immune TME and activated the transcription of immune networks both *in vitro* and *in vivo*. Specifically, we identified accumulation of effector cytotoxic lymphocytes and NK cells, and reductions in immunosuppressive Treg CD4^+^ cells. These changes were accompanied by a small but significant prolongation of survival *in vivo*. Furthermore, treatment also reduced the expression of the suppressive receptor CD206 on dendritic cells and macrophages, and blocked monocyte-to-macrophage differentiation in both the TME and peritoneal cavity. TAMs derive from the large population of CCR2^high^Ly6C^+^ inflammatory monocytes that constantly contributes to the pool, and Ly6C expression gradually reduces as TAMs differentiate within tumors (44). HKMT-1–005 treatment increased the abundance of Ly6C^+^ macrophages, suggesting that this epigenetic modifier may impede the differentiation of the monocyte precursor pool into fully differentiated TAMs.

The preclinical results presented here provide evidence that dual inhibition of G9A and EZH2 induces more robust chemokine induction than blockade of either methyltransferase alone. Recently, the co-dependence of EZH2 and G9A was established by Mozzetta and colleagues (36) and this has led to efforts to discover pharmacologic inhibitors that target both enzymes simultaneously, with HKMTI-1–005 being the described first (19). Curry and colleagues showed that treatment of the breast cancer cell line MDA-MB-231 with HKMTI-1–005 induced transcription of *SPINK1*, which did not occur when *EZH2* or *G9A* were individually knocked down (19). The interplay between EZH2 and G9A in regulating *CXCL10* transcription has also recently been observed in idiopathic pulmonary fibrosis by Coward and colleagues (45), further supporting our findings.

This work has generated interesting questions with regards to mechanism of action of G9A/EZH2 blockade that will need further investigation. First, our transcriptional and chromatin accessibility analyses were based on whole-tumor sequencing and therefore do not identify the cell type subjected to transcriptional modifications by HKMTI-1–005 treatment. Single-cell sequencing may help to delineate whether HKMI-1–005 acts primarily on tumor or immune cells *in vivo*. Second, the contribution of ERV-K retroelements to the immune responses following HKMTI-1–005 treatment warrants further exploration. Recent evidence suggests that ERVs can potentiate antitumor immunity when they are transcriptionally active (4, 46, 47) and that activation of evolutionary young elements is associated with innate immune responses (48). ERVK, an evolutionary young element, was activated following treatment with HKMTI-1–005 and, interestingly, antibodies against ERVK have been detected in the serum of patients with ovarian cancer (49). Similarly, our team has previously shown that expression of ERVK elements correlate with a transcriptome indicative of strong immune cell infiltration in TCGA ovarian carcinoma datasets (50) and that epigenetic manipulation of ERV expression by DNA methyltransferase inhibition can result in augmented immune cell killing of tumor cells *in vitro*. Recently, Steiner and colleagues mapped human ERVs at a locus-specific resolution, creating the platform whereby ERVs and their relation to immune response can be further explored in greater granularity (51). The downregulation of the macrophage receptor MARCO following HKMTI-1–005 treatment is also an intriguing finding; inhibiting MARCO reprogrammes macrophages to acquire an antitumor phenotype, inhibiting tumor cell growth (52). In the work presented here, we used a single ovarian cancer mouse model ID8, engineered with *Trp53*^−/−^ deletion, which represents the only universal genomic alteration in HGSC. This model faithfully recreates the intra-abdominal dissemination of HGSC with widespread peritoneal and omental deposits and formation of ascites, as commonly observed in human disease. Moreover, the models that we generated are now in widespread use and have supported critical studies on the nature of immune cell composition in the TME (53, 54). However, it will be important in future work to assess the influence of tumor genotype in the response to HKMTI-1–005 in light of recent data showing that BRCA1 deficiency drives inflammation that supports both immunoreactivity and immune resistance (55). However, we used a series of established human ovarian cancer cell lines that are representative of HGSC as well as primary ascites-derived cultures to reinforce our findings with the *Trp53*^−/−^ ID8 model.

Although the primary aim of our study was to investigate epigenetic regulation of the TME, one critical outstanding question is whether the increase in survival seen following HKMTI-1–005 treatment is driven by the changes in immune cell composition. Certainly, the doses utilized *in vitro* were noncytotoxic, but detailed evaluation would require depletion of multiple immune lineages as well as complex combination experiments that lie beyond the scope of this study. However, the results that we present support the hypothesis that dual blockade of G9A/EZH2 histone methyltransferases modulates the tumor immune microenvironment within the peritoneal cavity, confers a survival benefit in an aggressive murine model of HGSC and warrants further investigation towards clinical development.

## Authors' Disclosures

S. Spear reports grants from Imperial College London during the conduct of the study. P. Roxburgh reports grants, personal fees, and nonfinancial support from AstraZeneca and Tesaro/GSK; grants from Atrios, Immuntep, Iovance, Starpharma, Sierra Oncology, Replimune, Clovis, Athenex, Bayer, Forma Therapeutics, and PsiOxus outside the submitted work. M.J. Fuchter reports grants from EPSRC during the conduct of the study; personal fees from NK:IO Ltd. and grants from Apollo Therapeutics Ltd. outside the submitted work; also has a patent for WO2013140148 issued. R. Brown reports grants from Ovarian Cancer Action and grants from Imperial Confidence in Concept during the conduct of the study; also has a patent for PCT/GB2013/050689 Novel HKMT inhibitors issued. I.A. McNeish reports personal fees from Clovis Oncology, GSK/Tesaro, Roche, Epsila, Transgene, and Theolytics; grants and personal fees from AstraZeneca outside the submitted work. No disclosures were reported by the other authors.

## Supplementary Material

Supplementary Figure

Supplementary Data

Supplementary Table

Supplementary Table

Supplementary Table

Supplementary Table

Supplementary Data
