# Supplementary material for: Dual G9A/EZH2 Inhibition Stimulates Antitumor Immune Response in Ovarian High-Grade Serous Carcinoma
Source: Mol Cancer Ther. 2022 Feb 7;21(4):522–34. doi: 10.1158/1535-7163.MCT-21-0743 (PMC9377747; doi:10.1158/1535-7163.MCT-21-0743)
Supplement: Supplementary Figure [file mct-21-0743_supplementary_figures_s1_-_s8_supps1-s8.docx]

**Supplementary figures and legends**

**Table S1:** Library of epigenetic probes used in the drug-screening as per Structural Genomics Consortium (SGC, 2017).

**Table S2:** Primers used for single-gene RT-qPCR. All primers supplied by ThermoFisher.

**Table S3:** List of cytokines and chemokines in Qiagen array used *in vitro* in Figure 2D.

**Table S4:** Quality control of the Qiagen array used *in vitro* in Figure 2D.

**Table S5:** Normalisation analysis of the Qiagen array used *in vitro* in Figure 2D.

**Table S6:** Clinical characteristics and validation of primary ascites cultures from Figure 2F results.

**Table S7:** List of conjugated antibodies used for immunophenotyping by flow cytometry in Figure 5 and Figure 6.

**Table S8:** Full list of differentially expressed genes in HKMTI-1-005 treated tumours compared to vehicle

**Table S9:** Full list of differentially expressed ERV in HKMTI-1-005 treated tumours compared to vehicle

**Table S10:** Functional annotation of upregulated genes (RNAseq) overlapping with differential chromatin peaks (ATAC-seq) in HKMTI-1-005 treated tumours compared to vehicle

**Table S11:** Functional annotation of downregulated genes (RNAseq) overlapping with differential chromatin peaks (ATAC-seq) in HKMTI-1-005 treated tumours compared to vehicle

**Supplementary figures**

**Figure S1: Intra-abdominal tumour deposits in C57BL/6J mice following intraperitoneal inoculation with *Trp53^-/-^* ID8 cells**

(a) C57BL/6J mouse; omental deposit (rectangle) and peritoneal deposits (arrows) are sites of disease, 6 weeks after IP inoculation with 5x10^6^ *Trp53^-/-^* ID8 cells in 200 μl of PBS. (b) Porta hepatis deposit (circle) after part of liver removed.

**Figure S2: Drug response curves for SGC library.**

2x10^3^ ID8 *Trp53*^-/-^ cells were plated on day 0 in 384 well plates and treated with the SGC library on day 1, at concentrations 20 nM - 10 μM. Loss of viability on y axis as measured by DAPI stained nuclei in treated wells relative to DMSO controls (n=3 technical replicates).

**Figure S3: Western blot analysis following inhibition of G9a, EZH2 and combination G9a/EZH2**.

Western blot analysis for H3K9 methylation marks with the G9a inhibitor, UNC0642 (a) and G9a/Ezh2 inhibitor, HKMTI-1-005 (c) and H3K27me trimethylation marks for Ezh2 inhibitor, UNC1999 (b) and HKMTI-1-005 (d). Treatment duration was 48 hours. Histone H3 acted as a loading control. Protein electrophoresis was performed for each methylation mark and its respective loading control on separate gels, given that both the size of H3 and the size of the methylated mark were both approximately 17kDa.

Primary antibodies used: Anti-H3K27me3 (mouse, ab6001), Anti-H3K9me (rabbit, ab9045), Anti-H3K9me2 (mouse, ab1220), Anti-H3K9me3 (rabbit, ab176916), Anti-H3 (mouse, ab24834).

Secondary antibodies used: Anti-mouse (DAKO, P0448) and Anti-rabbit (DAKO, P0447).

**Figure S4: Cell viability assay (MTT) with HKMTI-1-005**

*Trp53^-/-^* ID8 cells treated with 1ng/ml of IFNγ or IFNγ plus variable doses of HKMTI-1-005. Experiment performed in three technical replicates and means compared with one-way ANOVA and Dunnett’s multiple comparisons. Error bars represent standard error of mean.

*p <0.05, ns= non-significant.

**Figure S5: Mouse weight during treatment**

Mouse body weight (n=24 per cohort) with either vehicle (1% Tween/3.6% DMSO in 0.9% NaCl IP bd) or HKMTI-1-005 (20mg/kg IP bd). Median weight was 19.8g ± 0.19 *vs* 20.1g ± 0.18, p=0.38, with vehicle and HKMTI-1-005, respectively. Unpaired t-test was used to compare means, bars show standard error of mean. IP: intraperitoneal, bd: twice daily.

**Figure S6: RNA sequencing- ImmuCC tool** (a) Immune cell distribution in the vehicle

(left panel) and treatment group (right panel) as per ImmuCC tool. (b) quantification of

(a) as percentages.

**Figure S7. Expression of CD206 on macrophages**. CD206 median fluorescence intensity (MFI) on macrophages isolated from porta hepatis (n=6 vehicle and n=7 HKMTI-1-005) and omentum (n=9 vehicle and n=8 HKMTI-1-005). Statistical significance was tested by unpaired *t*-test. *p<0.05

**Figure S8 Immunohistochemistry of omental tumours harvested immediately after HKMTI-1-005 treatment**. Tumours harvested immediately after completion of HKMTI-1-005 treatment (as per Fig 4a) were stained for CD3, NKp46 (NK cell marker) and FoxP3 by immunohistochemistry. We observed an increase in both CD3 (H-score 14.8 ± 1.8 *vs* 20.4 ± 2.8, p=0.09) and NKp46 staining (3.4 ± 0.4 *vs* 4.6 ± 0.9, p=0.08) and a decrease FoxP3 staining (13.1 ± 2.64 *vs* 9.6 ± 2.07, p=0.3).
