# Supplementary material for: Dual G9A/EZH2 Inhibition Stimulates Antitumor Immune Response in Ovarian High-Grade Serous Carcinoma
Source: Mol Cancer Ther. 2022 Feb 7;21(4):522–34. doi: 10.1158/1535-7163.MCT-21-0743 (PMC9377747; doi:10.1158/1535-7163.MCT-21-0743)
Supplement: Supplementary Data [file mct-21-0743_supplementary_methods_supp1.docx]

**Supplementary Methods**

*MTT cell viability assay*

MTT assay was performed on cells seeded at a density of 3x10^3^ cells (for the ID8 cell line) per well in 24-well Corning plates in 700 μl medium. Cells were treated with the indicated drug and for the desired duration (usually 48-72 hours). Thiazolyl blue tetrazolium bromide powder (Sigma Aldrich, M2128) was dissolved in PBS to a 5 mg/ml concentration (MTT reagent). At the desired endpoint, medium was aspirated and replaced with 700 μl MTT reagent diluted 1 in 10 with culture medium. Cells were then incubated for 4 hours at 37°C. During this 4-hour period, viable cells with active metabolism are expected to convert MTT reagent into formazan crystals. Crystals were then dissolved in 500 μl DMSO. Plates were gently mixed for 5 minutes, then transferred to an Infinite® 200 Tecan plate reader for analysis of absorbance (as a marker of cell viability) at 560 nm wavelength. Neat DMSO absorbance was measured in DMSO-only dedicated wells and subtracted from all other detected absorbances.

*Immunohistochemistry*

Immunohistochemical (IHC) staining for CD3, NKp46 and Foxp3 was performed on 4 μm formalin fixed paraffin embedded (FFPE) sections that had previously been heated in an oven at 60C^o^ for 2 hours. IHC staining for CD3 was performed on a Leica Bond Rx Autostainer and staining for NKp46 and Foxp3 was performed on an Agilent Autostainer Link48. The antibodies were used at previously optimised dilutions: CD3 (Abcam ab16669) at 1/100, NKp46 (R&D Systems AF2225) at 1/200 and FoxP3 (Cell Signalling 12653) at 1/200 dilution. The stained sections were then digitally captured on a Leica SCN400f slide scanner and image analysis was performed using HALO software (Indica Labs). Using the HALO software, firstly, non-malignant areas of tissue were manually excluded. Images were then analysed for the protein of interest using the CytoNuclear analysis tool v1.6. Images were then automatically scored using the histoscore method as described by Kirkegaard et al. This algorithm is embedded in HALO software and after grading the staining intensity as negative (0), weakly stained (1), moderately stained (2) and strongly stained (3), it uses the formula below to calculate a histoscore value ranging between 0 and 300:

sum of (1 x % cells stained 1) + (2 x % stained 2) + (3 x % stained 3).

*ATAC sequencing filtration strategy*

For the analysis of ATACseq results, a filtration strategy was devised whereby only those peaks that were present in 2/5 HKMTI-1-005 samples and in 3/6 control samples were considered to truly represent areas of open chromatin. These were 74,424/184,605 (40%) in the control samples and 273,774/ 487,409 (56%). Subsequently, all the overlapping peaks between this group of peaks were removed and a remaining 218,718/273,774 peaks (80%) in the HKMTI-1-005 group were further interrogated for the presence of genes. 15,636 unique genes with any length of overlap with these 218,718 peaks were found and further examined for overlap with RNA sequencing differentially expressed genes (DEGs), as depicted in volcano plot in **Fig 3D**.

*ERV sequencing analysis strategy*

To remove low-expressing ERVs from our sequencing data, we applied a filtration threshold whereby an ERV needed a count of at least n=10 in one or more samples (in either the vehicle or HKMTI-1-005 group) in order to be included in our analysis. The total number of ERVs detected was 61,184 but only 2,781 and 2,465 passed the above filtration threshold in the control and treatment groups, respectively. 2,118 ERVs were common to both groups and they were subjected to differential expression analysis using the DESeq2 package. Out of these 2,118 ERVs, 51 were differentially expressed at the 5% FDR threshold.

*Next generation sequencing*

DNA was extracted from 1-5×10^6^ cells using QIAmp DNA minikit (Qiagen, UK) according to the manufacturer's protocol. 50-200ng DNA was sheared with a Covaris LE220 focused-ultrasonicator (Covaris, Woburn, MA) to produce 100-200bp fragments. Libraries were generated using SureSelect XT standard protocol (Agilent Technologies, Santa Clara, CA) for low-input and FFPE samples. Analysis of *TP53* was performed using the Ampliseq platform on a HiSeq4000 system (Illumina, Cambridge, UK), using paired-end 125 bp protocols. The mean coverage was >7000×. FASTQ files were trimmed for adapters and aligned to reference human genome hg19 using Burrows-Wheeler Alignment (BWA-MEM) (1) and pre-processed using samtools and Picard to generate sorted BAM files (2). Somatic mutations were called using Mutect2 (GATK4.1.4.1) and Strelka (v1.0.14) (3) for single nucleotide variations (SNVs) and small insertions and deletions (indels) using default parameters. Mutations were annotated using Variant Effect Predictor (VEP) (version 1.5.3) (4). Somatic mutations were filtered by clinical significance (ClinVar, February 2021) with “benign” and “likely_benign” variants discarded.

*PAX8 immunocytochemistry*

Cells were pelleted by centrifugation and resuspended in 5ml Neutral buffered formalin for 1 hour at room temperature, then washed twice in PBS. The cell pellet was resuspended in 200µl liquified HistoGel™(Thermo Fisher Scientific, product code 12006679) and allowed to set. Processed cells were embedded in a molten wax cassette. 5µm sections were cut, deparaffinized and hydrated. Heat mediated antigen retrieval was performed in EDTA based pH 9.0 solution. Endogenous peroxidase was quenched with 3% hydrogen peroxide. The sections were incubated with mouse anti-PAX8 monoclonal antibody (1:50 dilution, Abcam, ab53490), followed by rabbit anti-mouse IgG and anti-rabbit IgG conjugated with polymeric horseradish peroxidase linker (Leica Bond Polymer Refine Detection, DS9800). DAB was used as the chromogen and the sections were then counterstained with haematoxylin and mounted with DPX. Staining was performed using a Leica BOND RX. Stained slides were scanned with NanoZoomer 2.0HT (Hamamatsu, Japan). NDP.scan 3.2.12 software was used for digital image acquisition and NDP.view2 software was used for image viewing.

**References**

1. Li H, Durbin R. Fast and accurate short read alignment with Burrows-Wheeler transform. Bioinformatics (Oxford, England) **2009**;25(14):1754-60 doi 10.1093/bioinformatics/btp324.

2. Sandmann S, de Graaf AO, Karimi M, van der Reijden BA, Hellstrom-Lindberg E, Jansen JH*, et al.* Evaluating Variant Calling Tools for Non-Matched Next-Generation Sequencing Data. Scientific reports **2017**;7:43169 doi 10.1038/srep43169.

3. Saunders CT, Wong WS, Swamy S, Becq J, Murray LJ, Cheetham RK. Strelka: accurate somatic small-variant calling from sequenced tumor-normal sample pairs. Bioinformatics (Oxford, England) **2012**;28(14):1811-7 doi 10.1093/bioinformatics/bts271.

4. McLaren W, Gil L, Hunt SE, Riat HS, Ritchie GR, Thormann A*, et al.* The Ensembl Variant Effect Predictor. Genome biology **2016**;17(1):122 doi 10.1186/s13059-016-0974-4.
